# The Catalytic Activity of Human REV1 on Undamaged and Damaged DNA

**DOI:** 10.3390/ijms25074107

**Published:** 2024-04-08

**Authors:** Anastasia D. Stolyarenko, Anna A. Novikova, Evgeniy S. Shilkin, Valentin A. Poltorachenko, Alena V. Makarova

**Affiliations:** 1 National Research Center “Kurchatov Institute”, 123182 Moscow, Russia; novannnov00@gmail.com (A.A.N.); shilkinevgeniy.chem@gmail.com (E.S.S.); poltora4enko@gmail.com (V.A.P.); 2 Institute of Gene Biology of the Russian Academy of Sciences, 119334 Moscow, Russia

**Keywords:** REV1, DNA polymerase, translesion DNA synthesis, AP site, cisplatin intrastrand crosslink, Hoogsteen interactions

## Abstract

Eukaryotic REV1 serves as a scaffold protein for the coordination of DNA polymerases during DNA translesion synthesis. Besides this structural role, REV1 is a Y-family DNA polymerase with its own distributive deoxycytidyl transferase activity. However, data about the accuracy and efficiency of DNA synthesis by REV1 in the literature are contrasting. Here, we expressed and purified the full-length human REV1 from *Saccharomyces cerevisiae* and characterized its activity on undamaged DNA and a wide range of damaged DNA templates. We demonstrated that REV1 carried out accurate synthesis opposite 8-oxoG and O^6^-meG with moderate efficiency. It also replicated thymine glycol surprisingly well in an error-prone manner, but was blocked by the intrastrand 1,2-GG cisplatin crosslink. By using the 1,N^6^-ethenoadenine and 7-deaza-adenine lesions, we have provided biochemical evidence of the importance for REV1 functioning of the Hoogsteen face of template A, the second preferable template after G.

## 1. Introduction

DNA damage can cause replication arrest which is deleterious for the living cell. DNA translesion synthesis (TLS) is a mechanism to bypass DNA lesions and protect cells against replication stress. An important role in TLS belongs to the low-fidelity inserter DNA polymerases Pol ι, Pol η or Pol κ (Y-family), extender DNA polymerase Pol ζ (B-family), and a key bifunctional REV1 protein. All of them function in humans, while only Pol ζ, Pol η and REV1 are conserved throughout eukaryotes [1]. 

REV1 belongs to the Y-family of DNA polymerases and functions both as a DNA polymerase and the integrator scaffold protein for the assembly of the translesome. REV1 can simultaneously interact with the monoubiqutinated PCNA processivity factor, one of the three Y-family polymerases (the choice depends on the lesion), and several subunits of extender Pol ζ (for a review, see [2]). Multiple protein–protein interactions ensure the coordination of the replication enzymes and accomplish the switch from a high-fidelity replicative polymerase to translesion DNA polymerases and from a less processive inserter translesion DNA polymerase to the extender polymerase Pol ζ. Mutations in the *REV1* gene blocking protein–protein interactions disturb TLS to a much greater extent than the substitutions of the catalytic residues, suggesting that the primary role of REV1 in TLS is structural or regulatory [3,4,5,6]. 

As a DNA polymerase, REV1 possesses weak DNA polymerase activity, usually referred to as dCTP transferase [7]. Its catalytic activity is driven by a unique protein–template mechanism, when the incoming dCTP and the DNA template base do not directly pair with each other [8]. Instead, dCTP pairs with Agr357 of human REV1, while template G pairs via its Hoogsteen face with the main chain amides of His774 and Gly775 of the G-loop of the PAD domain of REV1, in addition to being evicted from the DNA helix by Leu358. 

REV1 is one of the few polymerases capable of efficiently replicating AP sites in vitro [7,9,10] and in vivo, where its catalytic activity has been shown to be required for the bypass [11,12,13]. Also, the catalytic activity of REV1 is required for 1,N^6^-ethenoadenine (εA) bypass [12,14] and plays a role in G4 DNA replication, contributing to incorporation of about 50% of C opposite G [15]. Its catalytic activity has a role in somatic hypermutation of immunoglobulin genes in B-lymphocytes, leading to C to G transversions [16]. Thus, REV1 enzymatic function is biologically relevant and is required for TLS in a lesion-specific manner. Moreover, REV1 possesses weak 5′-deoxyribophosphate lyase activity and possibly might substitute for Pol β (as a backup enzyme) in some base excision repair reactions [17]. Alterations in *REV1* expression and/or catalytic activity promote mutagenesis and carcinogenesis and can modulate the effect of chemotherapy treatment [18,19,20,21]. Thus, REV1 is a key protein for protecting genome stability.

The catalytic activity of human REV1 is not fully characterized. While isolation of the full-length active REV1 of *S. cerevisiae* is described in the literature and is not methodologically difficult [5,7,22,23,24,25,26], the study of human REV1 is limited by the complexity of isolating full-length protein preparations of high quality. Since human Y-family polymerases are prone to degradation of the C-terminus [27], many biochemical studies have been carried out with the catalytic core of REV1 [24,28,29,30,31,32,33,34,35,36,37]. However, the activity of the catalytic core or truncated REV1 protein can slightly differ. Moreover, biochemical studies of the catalytic core also do not allow researchers to analyze the TLS activity in the presence of accessory proteins and other TLS DNA polymerases.

In this work, we purified full-length human REV1 from *S. cerevisiae* and characterized its catalytic activity on undamaged DNA templates and opposite a wide range of DNA lesions. We showed that among G templates with DNA damage, 8-oxoG and O^6^-meG lesion were bypassed with high accuracy and moderate efficiency, but the 7-deazaG decreased and 1,2-GG cisplatin intrastrand crosslink (1,2-GG CisPt CL) blocked REV1 activity. Among the adducts of template A, the second most favorite template of REV1, Watson–Crick-face-disrupting εA was replicated by REV1 far more efficiently than the Hoogsteen-face-impairing 7-deazaA, demonstrating error-prone behavior. Among the lesions of pyrimidines, the least favorite templates of REV1, thymine glycol (TG) was surprisingly well replicated in an error-prone manner. 1,2-GG CisPt CL, TG and the 7-deaza-purine analogs were studied for the first time.

## 2. Results

### 2.1. Activity of REV1 on Undamaged DNA and the AP Site

Human REV1 is a single-subunit protein of 138 kDa. Although several research groups have purified the full-length human REV1 from *E. coli* [27,38,39] and yeast [9], many laboratories have used the catalytic core of REV1 for biochemical studies [24,28,29,30,31,32,33,34]. 

**Figure 1:**
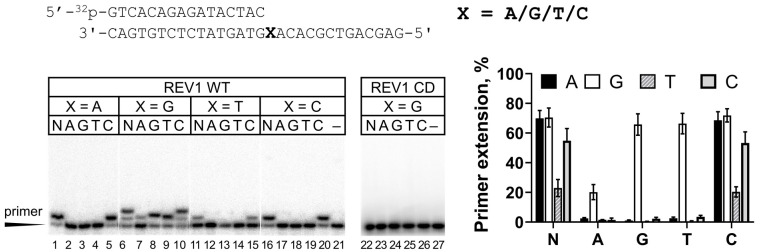
REV1 activity on DNA containing undamaged A, G, T or C. Primer extension reactions were carried out in the presence of the wild-type REV1 protein (WT) or its catalytically inactive variant (CD) and all four dNTPs (N) or individual nucleotide substrates (A—dATP, G—dGTP, T—dTTP and C—dCTP); “–”—control reactions without the enzyme. The mean values of primer extension and standard errors are indicated.

**Figure A1:**
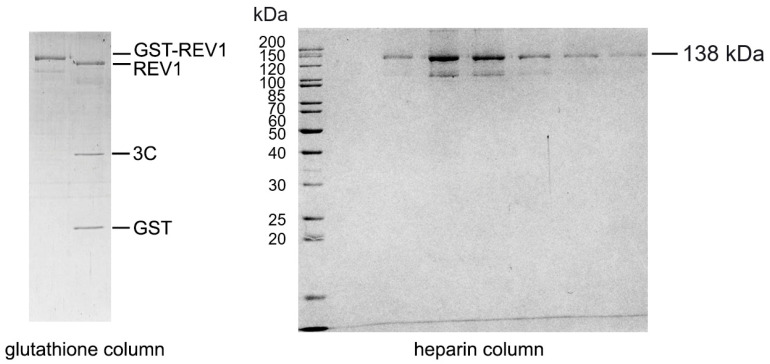
Protein preparation of human REV1: 10% SDS-PAGE and Coomassie brilliant blue G-250 staining. Several faint bands are observed below the band corresponding to the human REV1 protein (138 kDa) which are characteristic of the C-terminal degradation products of Y-family polymerases.

In our laboratory, expression of the full-length human REV1 in *E. coli* provided highly proteolyzed preparations with low catalytic activity. Purification of human REV1 from yeast cells yielded low amounts of protein (up to 70 µg from 24 L of culture) (Appendix A, Figure A1), but these preparations demonstrated high catalytic activity (Figure 1). The absence of impurities affecting the activity of REV1 was verified using the catalytically inactive REV1 variant (REV1-CD) purified by the same protocol (Figure 1, lanes 23–27). REV1 incorporated nucleotides opposite all undamaged DNA templates at a +1 position, with the lowest catalytic activity observed opposite T, as judged from primer extension analysis (Figure 1, lanes 11–15). As expected, REV1 apparently incorporated only dCMP opposite templates A, T and C (Figure 1, lanes 5, 15 and 20).

Template G is the preferable DNA substrate for REV1. dCMP incorporation was very efficient and continued to position +2, which was frequently observed on the G template in the literature [7,24,38]. Efficient synthesis in position +2 can possibly be explained by the fact that the G template continued with A, known to be the second preferable substate for REV1 [7,10,27,39], also readily replicated by dCMP incorporation. Opposite template G, human REV1 incorporated with high efficiency not only dCMP but also non-complementary dGMP and dTMP (Figure 1, lanes 8 and 9) and incorporated dAMP with low efficiency (Figure 1, lane 7), showing the lowest accuracy of DNA synthesis on undamaged DNA templates. According to the literature, REV1 incorporates non-complementary dNMP opposite G with 1000–10,000-fold reduced efficiencies compared to the complementary dCMP [7,22,27,33,40]. The relatively high incorporation of dGMP and dTMP opposite G in this work is somewhat surprising and possibly can be explained by the effect of the DNA sequence context. The relatively efficient incorporation of non-complementary nucleotides was also observed in others studies [10,32,34]. 

**Figure 2:**
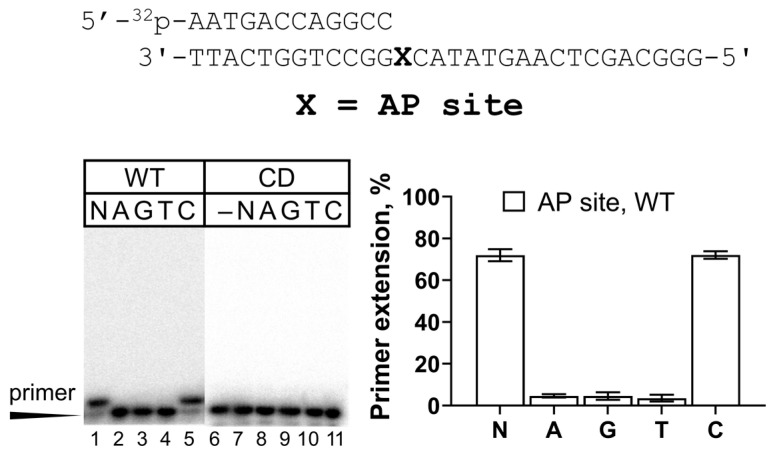
REV1 activity on DNA containing the AP site. Primer extension reactions were carried out in the presence of the wild-type REV1 protein (WT) or its catalytically inactive variant (CD) and all four dNTPs (N) or individual nucleotide substrates (A—dATP, G—dGTP, T—dTTP and C—dCTP); “–”—control reactions without dNTPs. The mean values of primer extension and standard errors are indicated.

The AP site is another classical DNA substrate of REV1. It has been demonstrated both in vitro [7,9,10] and in vivo [11,12,13] that REV1 is involved in TLS opposite the AP site. In agreement with the literature data, REV1 efficiently incorporated dCMP opposite the AP site (Figure 2).

### 2.2. REV1 Activity on DNA with G Lesions

**Figure 3:**
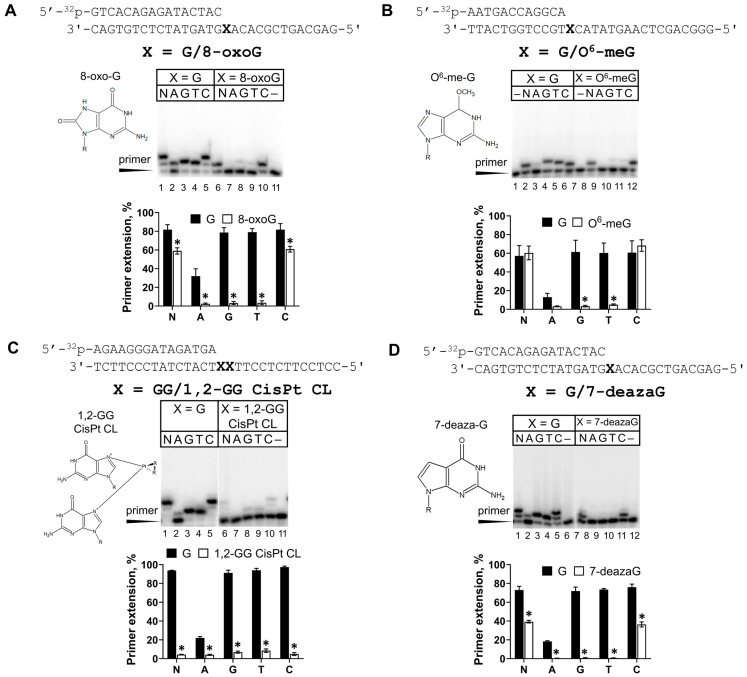
REV1 activity on DNA containing 8-oxoG (**A**), O^6^-meG (**B**) and 1,2-GG CisPt CL (**C**) and the 7-deazaG analog (**D**). Primer extension reactions were carried out in the presence of the wild-type REV1 and all four dNTPs (N) or individual nucleotide substrates (A—dATP, G—dGTP, T—dTTP and C—dCTP); “–”—control reactions without dNTPs. The mean values of primer extension and standard errors are indicated; *p*-values < 0.05 are shown by *.

REV1 demonstrates efficient and accurate activity opposite many types of non-bulky and bulky G lesions (e.g., see [38]). However, literature data for a number of lesions are contrasting. For example, the full-length human REV1 incorporated dCMP opposite 8-oxoG not more than 10-fold less efficiently compared to undamaged G [27,41], while the catalytic core of human REV1 was 60–90-fold less efficient opposite 8-oxoG compared to control G [24,29]. *S. cerevisiae* REV1 was less potent, demonstrating a 370–1000-fold reduction in the activity opposite 8-oxoG, shown by different methods [7,42]. Our preparation of the full-length human REV1 incorporated dCMP opposite 8-oxoG with moderate efficiency in the primer extension assay (Figure 3A). In particular, REV1 incorporated dCMP opposite 8-oxoG 366-fold less efficiently than opposite undamaged G (Table 1), showing a marked increase in K_M_ for dCMP. The incorporation of trace amounts of non-complementary dGMP and dTMP was also observed (Figure 3A). Overall, our data are in agreement with the results obtained for the yeast enzyme [7]. 

REV1 also demonstrated accurate and efficient synthesis opposite O^6^-meG (Figure 3B). The 24-fold reduction in efficiency of dCMP incorporation opposite O^6^-meG compared to G was also accompanied by increased K_M_, but no k_cat_ change (Table 1). This result is in agreement with the 16–39-fold reduction in dCMP incorporation opposite O^6^-meG for human and yeast REV1 [7,38]. In our work, REV1 incorporated very small amounts of non-complementary dGMP and dTMP opposite the O^6^-meG lesion, in agreement with [38], where it was shown only by kinetic measurements. The control G for O^6^-meG (in GC with the 12-mer primer) was replicated 2-fold less efficiently than the control G for 8-oxoG (in GA with the 16-mer primer), the latter also having a significant +2 extension signal, while in the case of O^6^-meG, the +2 signal was low. This can possibly be explained by the differences in DNA substrate structure such as the length of the DNA primer or DNA sequence context. In particular, A as the +2 template nucleotide might be more preferable than C for REV1, or the “long” 16-mer primer might contribute to DNA binding better than the “short” 12-mer primer.

In this work, we also, for the first time, analyzed the activity of REV1 opposite the 1,2-GG cisplatin crosslink and demonstrated that REV1 was almost fully blocked by this lesion (Figure 3C). 

Along with G lesions, we also analyzed for the first time the 7-deazaG analog with impaired Hoogsteen base pair formation. In line with the influence of O^6^-meG and 8-oxoG, 7-deazaG inhibited REV1 catalytic activity, and very small amounts of non-complementary dGMP and dTMP were observed (Figure 3D). 

### 2.3. REV1 Activity Opposite εA and 7-DeazaA

**Figure 4:**
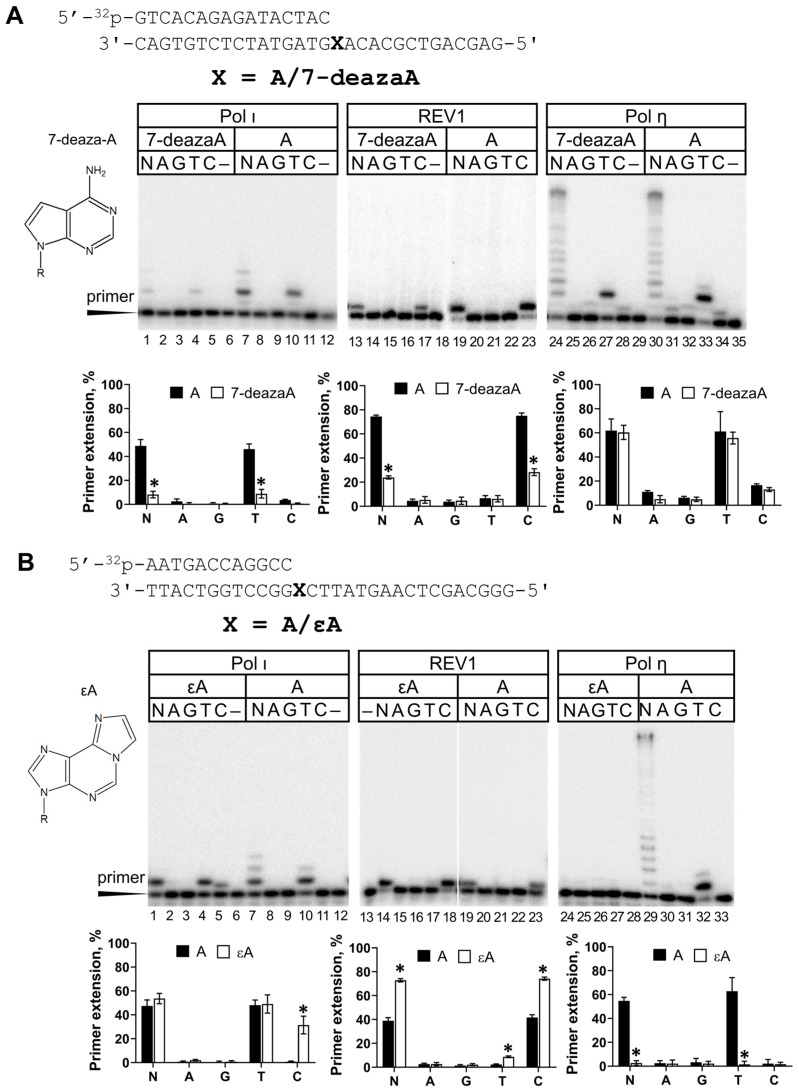
REV1 activity on Hoogsteen-blocking 7-deazaA (**A**) and Watson–Crick-blocking εA (**B**) lesions. Primer extension reactions were carried out in the presence of the wild-type REV1 and all four dNTPs (N) or individual nucleotide substrates (A—dATP, G—dGTP, T—dTTP and C—dCTP); “–”—control reactions without dNTPs. The mean values of primer extension and standard errors are indicated; *p*-values < 0.05 are shown by *.

The second favorite substrate after G for REV1 is template A. We continued the REV1 study with the Hoogsteen-plane-impairing 7-deazaA analog and the Watson–Crick-base-pair-impairing 1,N^6^-ethenoadenine (εA) lesion. For comparison, we tested the activity of Y-family Pol ι and Pol η supporting Hoogsteen and Watson–Crick interactions, respectively (Figure 4).

The activity inhibition of REV1 opposite 7-deazaA was more prominent compared to 7-deazaG (Figure 4A). Steady-state kinetics experiments demonstrated that REV1 incorporated dCMP opposite 7-deazaA with a 39-fold reduction in efficiency compared to A (Table 1). This effect was caused by both decreased affinity to dCTP (higher K_M_) and decreased reaction velocity (lower k_cat_). Even though REV1 was not fully inhibited opposite 7-deazaA and 7-deazaG analogs blocking Hoogsteen interactions, these data provide biochemical evidence for the importance of the Hoogsteen plane for REV1 catalytic activity, similarly to Pol ι (Figure 4A).

Unlike Pol η, REV1 demonstrated efficient but error-prone εA bypass, suggesting that REV1 does not require an intact Watson–Crick face of the template nucleotide, again resembling Pol ι (Figure 4B). Opposite template εA, REV1 was 54-fold more efficient than opposite the control A. This effect was accompanied by a dramatic difference in K_M_ for the undamaged and damaged substrate without a marked k_cat_ change (Table 1). Our data contrast with the results of Zhang et al., who demonstrated that human REV1 is only 1.3-fold more efficient opposite εA compared to A [27], but are in agreement with the very efficient εA bypass by yeast REV1 [43]. The authors suggested that incorporation opposite template A may be much less favorable than opposite εA due to the absence of the N^6^ hydrogen bond donor in εA, which in A would sterically hinder the binding of the G-loop of REV1, which is adapted to interact with the Hoogsteen edge of G preferred by REV1. Interestingly, we also observed a significant difference in K_M_ for dCMP incorporation opposite template A in control DNA substrates for εA and 7-deazaA which is possibly caused by the different DNA sequence context (A vs. C at +2 template position) and/or primer length (12 nt vs. 16 nt).

### 2.4. Activity of REV1 Opposite TG

**Figure 5:**
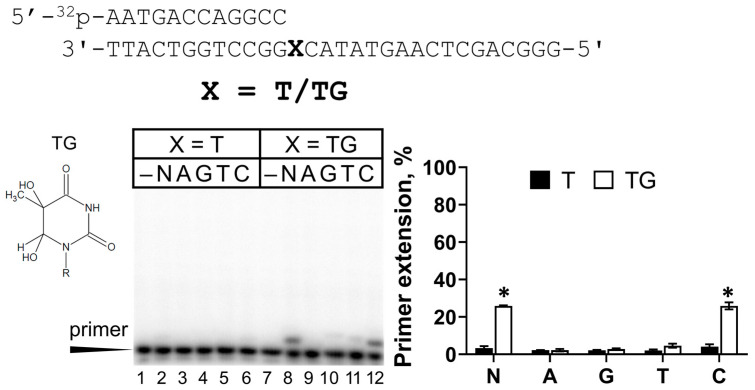
REV1 activity on DNA with TG. Primer extension reactions were carried out in the presence of the wild-type REV1 and all four dNTPs (N) or individual nucleotide substrates (A—dATP, G—dGTP, T—dTTP and C—dCTP); “–”—control reactions without enzyme. The mean values of primer extension and standard errors are indicated; *p*-values < 0.05 are shown by *.

Thymine glycol (TG) is a common DNA lesion caused by oxidative stress. This lesion has never been studied with human REV1. Among all undamaged DNA templates, REV1 possessed the lowest activity opposite template T (Figure 1). Surprisingly, we demonstrated that REV1 bypassed the TG lesion much more efficiently than the control T (Figure 5). In particular, REV1 incorporated dCMP opposite TG 1719-fold more efficiently, which was accompanied by a 300-fold K_M_ reduction (Table 1). Very weak incorporation of dGMP and dTMP was also observed. The control T for TG (in TC context with the 12-mer primer) was replicated significantly less efficiently than undamaged T, as shown in Figure 1 (in TA context with the 16-mer primer), again suggesting the role of DNA substrate structure.

Previously, the activity opposite TG was analyzed by primer extension analysis for mouse REV1 [44]. Mouse REV1 bypassed TG in the sequence context with two GGs at the +2-+3 positions and the authors hypothesized that Rev1 could rearrange the template and skip the TG lesion to incorporate two dCMPs opposite the next two template Gs. In this work, we demonstrated that REV1 carries out efficient TLS in the DNA sequence context (TG)C, suggesting that REV1 directly incorporates dCMP opposite the TG lesion.

## 3. Discussion

Isolation of the full-length active yeast REV1 is described in the literature and does not present significant methodological difficulties [7,22,23,24,25,26]. However, research on the properties of human REV1 is limited by the difficulty of purification of a protein of good quality. Isolation of human recombinant REV1 from *E. coli* provides relatively high protein yields (14–68 µg per 1 L) [27,38,39]. However, the purification of human recombinant enzymes from eukaryotic expression systems might provide some advantages over bacterial producers (such as correct folding and post-translational modifications). In the work [9], the partially purified preparation and the preparation purified to apparent homogeneity of human REV1 fused with the 6XHis tag were isolated from yeast. The protein possessed high catalytic activity (14.5 nM REV1 in reactions), but the partially purified preparation was used in most assays. Our work extends the list of purification methods of REV1 from eukaryotic producers and provides more information about the catalytic activity of human REV1. Several DNA lesions were analyzed with human REV1 for the first time.

The purified protein demonstrated classical properties of a dCMP transferase and was active in bypassing a number of earlier studied DNA lesions. It predominantly incorporated dCMP opposite the AP site, in accordance with the previously published results [9,27,37,39,45]. REV1 is known to efficiently incorporate complementary dCMP opposite many types of non-bulky and bulky G lesions, including adducts of the Watson–Crick plane in position N*^2^* [27,33,38,46] and adducts of the Hoogsteen plane in position 6 [38] and in position 8 [30,32]. Indeed, we observed accurate incorporation of dCMP by human REV1 opposite 8-oxoG and O^6^-meG lesions. Our data on the moderate TLS activity of the full-length human REV1 opposite 8-oxoG (366-fold lower efficiency compared to undamaged G) resemble earlier data in yeast [7]. This is somewhat different from the data on the full-length human REV1 preparation (2.6-fold inhibition) [27]. 

Our data on the TLS activity of REV1 opposite O^6^-meG are in agreement with the previous literature, showing REV1 to be accurate and efficient in its bypass in vitro [38]. However, REV1 was found not to be required for O^6^-meG bypass and mutagenesis in human cells [47]. Unlike 8-oxoG and O^6^-meG lesions, human REV1 was blocked opposite the 1,2-GG CisPt CL formed by a popular chemotherapy agent cisplatin, possibly due to steric hindrance. This is the first GG intrastrand crosslink studied with REV1 and the second intrastrand crosslink overall, with the TT dimer being the first. The UV-induced TT dimer also inhibited REV1 [23,27]. 

7-deazaA and 7-deazaG are artificial lesions constructed to monitor the effect of Hoogsteen face damage on DNA polymerase function. These modifications caused a considerable decrease in the activity of human REV1. The REV1 protein is known to recognize the G template nucleotide via its Hoogsteen edge, evident from the corresponding crystal structure [8]. The effect of 7-deazaG can be explained by the involvement of the Hoogsteen edge of template G in contact with the G-loop of the active site of REV1 [8,48]. The involvement of the Hoogsteen face of template A, the second favorite template of REV1, in base pairing is yet to be demonstrated structurally. We provided biochemical evidence that the Hoogsteen edge of template A is important for REV1 functioning, suggesting that the binding of template G and A in the active site of REV1 is similar (e.g., A is located in the enzyme’s binding pocket in the same conformation).

In contrast, Watson–Crick face disruption of A did not inhibit human REV1. Unlike most human DNA polymerases which are blocked by εA (except for Pol ι), REV1 bypassed the εA lesion even more effectively than the control A. Our data support the previous biochemical observations [27,43] and are in agreement with evidence of the catalytic role of REV1 in the bypass of εA in vivo in the yeast model [12,14]. 

The main structural feature of REV1 is the looping out of the template G nucleotide during DNA synthesis into the pocket formed by the enzyme. Thus, alterations in REV1 activity are often explained by disruptions in template nucleotide localization within the pocket. Indeed, intact templates A, T or C are much poorer substrates for REV1 since they are unable to form interactions specific for the G base. As suggested [43], the Hoogsteen edge of the A base contains the N^6^ amino group, which is an H-bond donor (while the O^6^ atom of G is an H-bond acceptor) and is likely to sterically hinder the binding of the unique G loop, reducing REV1 activity. Unlike the adenine base, the exocyclic N^6^ atom in εA lacks hydrogen bond donor properties, which partially restores REV1 activity to a level closer to that of template G.

Remarkably, we also demonstrated that human REV1 incorporated dCMP opposite TG more efficiently than opposite undamaged T. It was shown that human, mouse and yeast REV1 also bypass dU better than T [10,23,27]. Moreover, REV1 incorporated dCMP opposite hmC more efficiently than opposite mC (Shilkin et al., in preparation). Thus, it is likely that the addition of hydroxyl groups and the removal of the CH3 group of T can be beneficial for REV1 replication.

An overall general interesting observation regarding our kinetic steady-state data showed that substrates with longer primers and, in agreement with data for mouse REV1 protein [10], A (vs. C) at the +2 template positions are replicated more efficiently. Another conclusion is that most DNA lesions tested in the study affected K*_M_* for dCTP. This was previously discussed for the yeast enzyme, while the human enzyme demonstrated both K*_M_* and k*_cat_* changes [28,42].

## 4. Materials and Methods

### 4.1. Protein Purification

Four overlapping 960–980 bp DNA fragments encoding the human REV1 gene (REV1S isoform) were chemically synthesized (GeneCust company, Dudelange, Luxembourg) and cloned into the pRS424 yeast expression plasmid under the GAL1-10 promoter using the Gibson assembly method. REV1 fused with the N-terminal GST-tag was purified as described for yeast REV1 and Pol ζ [49,50] with modifications (Appendix A). Harvested yeast cells were resuspended in 2× lysis buffer and frozen dropwise in liquid nitrogen. The yeast “popcorn” was mechanically disrupted with dry ice in a laboratory blender and additionally sonicated after melting. DNA was removed by the polyethyleneimine precipitation procedure and the total protein from the clarified cell lysate was precipitated with 0.3 g/mL ammonium sulfate. REV1 was purified by glutathione affinity chromatography, followed by GST-tag removal with 3C precision protease. REV1 was separated from the GST-tag and protease by heparin–sepharose chromatography, dialyzed against storage buffer, aliquoted and stored at −80 °C. The catalytically dead variant of REV1 with substitutions D569A and E570A was obtained by site-directed mutagenesis and purified as the wild-type protein. Human Pol ι and Pol η were purified from *S. cerevisiae* cells, as previously described [51,52].

### 4.2. DNA Substrates for the Primer Extension Assay

To obtain DNA substrates for the primer extension reactions, ^32^P-labeled primers were annealed to the corresponding unlabeled template oligonucleotides. DNA templates with the AP site (a tetrahydrofuran analog), εA and O^6^-meG were purchased from Trilink BioTechnologies, Inc. (San Diego, CA, USA). DNA templates with 8-oxoG, thymine glycol, 7-deazaA and 7-deazaG were purchased from The Midland Certified Reagent Company (Midland, TX, USA) [53]. Preparation of the 1,2-GG CisPt CL was carried out in our laboratory previously [54]. Unmodified primers and the undamaged template DNA oligonucleotides were synthesized by Syntol and Evrogen (Moscow, Russia). The sequences of the oligonucleotides used in this study are shown in Table 2.

To prepare DNA substrates, the primers (Pr16, Pr15, Pr12 (ending with GCC) and Pr12 (ending with GCA)) were 5′-labeled with [γ-^32^P]-ATP by T4 polynucleotide kinase (SibEnzyme, Novosibirsk, Russia) for 1 h at 37 °C, with subsequent inactivation at 75 °C for 10 min. The primers were annealed to the corresponding unlabeled template oligonucleotides at a molar ratio of 1:1.1 in 100 mM NaCl by heating to 97 °C and slow cooling to 4 °C for 3.5 h.

### 4.3. DNA Polymerase Reactions for the Primer Extension Assay

Primer extension assays were performed in 10–20 µL reactions containing 20 nM ^32^P-labeled oligonucleotide substrate, 30 mM HEPES at pH 7.6, 50 mM NaCl, 10 mM MgCl_2_, 100 µg/mL BSA, 1 mM DTT, 8% glycerol and 5 nM of REV1 protein (2.5 nM for O^6^-meG and TG). Reactions were prepared on wet ice, started with the addition of 50 μM dNTPs, and incubated at 37 °C for 5 min. The reactions were stopped by the addition of an equal volume of 2× loading buffer (20 mM EDTA, 0.001% bromophenol blue and 96% formamide) and heated for 5 min at 95 °C. The reaction products were resolved on 21% (for 16- and 15-mer primers) or 23% (for 12-mer primers) polyacrylamide gels with 8 M urea, visualized on Typhoon 9400 (GE Healthcare Inc., Chicago, IL, USA) and analyzed with ImageQuant software Version 5.2 (Molecular Dynamics Inc., Sunnyvale, CA, USA). All experiments were repeated two to four times. The percentage of the extended primer (including all DNA bands) was calculated for each reaction; the mean values of primer extension with the standard errors of mean are shown in the figures. The data were compared using Student’s *t*-test and the differences were considered statistically significant at a *p*-value < 0.05.

### 4.4. Steady-State Kinetics Analysis of dNMP Incorporation

To quantify the incorporation of individual dCMPs opposite DNA lesions, we varied the dCTP concentration from 0.001 to 3000 μM in the reactions with 20 nM DNA substrate and 1 nM (for O^6^-meG and 8-oxoG) or 2.5 nM (for 7-deazaA, εA and TG) REV1. The reactions were incubated for a time between 45 sec and 9 min (depending on the lesion) to ensure that less than 20–40% of the primer was utilized at the maximum dCTP concentration. Calculations were done using GraphPad Prism software version 8.0.1 (GraphPad Software LLC, Boston, MA, USA). The data were fit to the Michaelis–Menten equation V = (V_max_ × [dNTP])/(K_M_ + [dNTP]), where V and V_max_ are the observed and the maximum rates of the reaction (in percentages of utilized primer per minute), respectively, and K_M_ is the apparent Michaelis constant. k_cat_ is Vmax divided by the protein concentration in the reaction. The calculated apparent K_M_ and k_cat_ parameters were used to determine the catalytic efficiency (k_cat_/K_M_). All experiments were performed three times.

## Figures and Tables

**Table 1:** Steady-state kinetics analysis of REV1 dCMP incorporation opposite various DNA lesions. F_inc_ was calculated as (k_cat_/K_M_)^lesion^/(k_cat_/K_M_)^control^. SEM for 3 replicates is shown. Decrease or increase in efficiency caused by the lesion relative to its control substrate is indicated by arrows (↓ or ↑, respectively).

Template Nucleotide	k_cat_, min^−1^	K_M_, µM	k_cat_/K_M_	F_inc_
G	4.86 ± 0.40	0.04 ± 0.01	133.29 ± 32.53	1
8-oxoG	3.20 ± 0.57	10.13 ± 3.02	0.36 ± 0.08	2.7∙10^−3^ (↓366 fold)
G	3.97 ± 0.38	0.06 ± 0.01	68.66 ± 0.61	1
O^6^-meG	7.22 ± 1.74	2.92 ± 1.21	2.87 ± 0.65	4.2∙10^−2^ (↓24 fold)
A	3.49 ± 0.11	10.60 ± 1.92	0.35 ± 0.07	1
7-deazaA	0.51 ± 0.05	56.81 ± 5.01	0.009 ± 0.001	2.6∙10^−2^ (↓39 fold)
A	1.87 ± 0.25	72.02 ± 11.69	0.03 ± 0.01	1
εA	3.19 ± 0.20	2.18 ± 0.27	1.53 ± 0.28	54 (↑54 fold)
T	0.09 ± 0.01	301.73 ± 27.45	(2.88 ± 0.07) × 10^−4^	1
TG	0.53 ± 0.06	1.08 ± 0.06	0.50 ± 0.06	1719 (↑1719 fold)

**Table 2:** Oligonucleotide pairs used in this study.

1	Primer 16: 5′-^32^P-GTCACAGAGATACTAC-3′ Template: 3′-CAGTGTCTCTATGATG**X**ACACGCTGACGAG-5′ where **X** = A, G, T, C, 8-oxoG, 7-deazaG and 7-deazaA
2	Primer 12 (GCC): 5′-^32^P-AATGACCAGGCC-3′ Template: 3′-TTACTGGTCCGG**X**CTTATGAACTCGACGGG-5′ where **X** = A and εA
3	Primer 12 (GCC): 5′-^32^P-AATGACCAGGCC-3′ Template: 3′-TTACTGGTCCGG**X**CATATGAACTCGACGGG-5′ where **X** = T, TG, and AP site (a tetrahydrofuran analog)
4	Primer 12 (GCA): 5′-^32^P-AATGACCAGGCA-3′ Template: 3′-TTACTGGTCCGT**X**CATATGAACTCGACGGG-5′ where **X** = G and O^6^-meG
5	Primer 15: 5′-^32^P-AGAAGGGATAGATGA-3′ Template: 3′-TCTTCCCTATCTACT**XX**TTCCTCTTCCTCC-5′ where **XX** = GG, and 1,2-GG CisPt CL

## Data Availability

No new data were created.

## Appendix A. Cloning and Purification of Human REV1

The REV1 isoform 2 (REV1S) [37] missing the Ala479 in the insertion I2 of the Fingers domain and containing the Val138Met (rs3087403, MAF = 0.24–0.4) and Phe257Ser (rs3087386, MAF = 0.45–0.55) substitutions was used in this study. The nucleotide sequence of the human *REV1* gene was optimized according to Dapcel algorithms (USA) for expression in *E. coli*. Four fragments of the *REV1* gene in the range of 960–980 bp, containing 30 nt long overlapping regions, were chemically synthesized by the GeneCust company (Laboratoire de Biotechnologie du Luxembourg, Dudelange, Luxembourg). The fragments were assembled and cloned into the pGEX-6P-1 bacterial and yeast pRS424 expression vectors in frame with the glutathione transferase gene by the Gibson Assembly Master Mix protocol (NEB, Ipswich, MA, USA, catalog # E2611S).

The production of human REV1 was performed with the BJ2168 strain of *S. cerevisiae*. After transformation with the pRS424-REV1 plasmid, yeast colonies were selected intryptophan-free minimal SCG medium and used to inoculate two 300 mL starter culture flasks with SCR liquid medium (with raffinose). Cells were grown at 30 °C and 200 rpm for 24 h and then inoculated into 12 liters of SCR medium (at a ratio of ~1:50) and grown for 24 h. Then, 12 L of YPD-rich medium was added, the culture was grown for 9 h and expression was induced by adding 1% galactose. After 7 h, cells were harvested by centrifugation at 4 °C (typically 200 g), washed with ice-cold water and resuspended in 2× lysis buffer (600 mM KCl, 100 mM HEPES at pH 7.4, 38 mM K_2_HPO_4_/KH_2_PO_4_ at pH 7.4, 4% glycerol, 1 mM DTT, 1 mM EDTA at pH 8, 0.01% NP-40, 0.5 mM PMSF, 8 μM pepstatin A, 10 μM leupeptin and 2.5 mM benzamidine), frozen dropwise in liquid nitrogen and stored at −80 °C.

Cell disruption was performed mechanically with dry ice in a laboratory blender (Waring, Stamford, CT, USA). The melted cell mixture was moved to a cold room and PMSF, DTT and glycerol were added to the cell lysate to achieve final concentrations of 1 mM, 2 mM and and 8%, respectively. Ammonium sulfate was added in small portions to achieve a final concentration of 190 mM and the mixture stirred on a magnetic stirrer until complete dissolution (about 20 min). This mixture was sonicated (for DNA fragmentation) on a SONICS Vibracell unit (Sonics & Materials, Newtown, CT, USA) for 4 min (pulse on 1.5 s, pulse off 4.5 s and amplitude 50%) and DNA was precipitated by 0.45% polyethyleneimine (PEI) (10% PEI was added dropwise under constant stirring for 20 min). Next, the lysate was clarified by centrifugation at 11,000 rpm for 20 min at 4 °C. Total soluble proteins were precipitated from the supernatant by adding ammonium sulfate powder to 0.31 g per 1 mL of cell lysate under constant stirring at 4 °C. After centrifugation, the precipitate was frozen in liquid nitrogen and stored at −80 °C.

The protein precipitate was resuspended in 300 mL of binding buffer (300 mM KCl, 30 mM HEPES at pH 7.4, 20 mM K_2_HPO_4_/KH_2_PO_4_ at pH 7.4, 8% glycerol, 1 mM DTT, 0.01% NP40, 0.05% Tween20, 0.5 mM PMSF, 8 μM pepstatin A, 10 μM leupeptin and 2.5 mM benzamidine) and incubated with 1–2 mL of glutathione–sepharose resin (GE Healthcare, USA) for 3 h on ice with rotation at 100 rpm. The resin was transferred to a 10 mL Bio-Rad column and washed with several buffers to increase the pH and decrease the salt content: buffer 1—600 mM KCl, 30 mM HEPES at pH 7.6, 20 mM K_2_HPO_4_/KH_2_PO_4_ at pH 7.6, 8% glycerol, 1 mM DTT, 0.5 mM EDTA at pH 8, 0.01% NP-40 and 0.05% Tween20; buffer 2—400 mM KCl, 30 mM HEPES at pH 7.8, 15 mM K_2_HPO_4_/KH_2_PO_4_ at pH 7.8, 8% glycerol, 1 mM DTT, 0.5 mM EDTA at pH 8, 0.01% NP-40, 0.05% Tween20, 0.5 mM ATP, 5 mM MgCl_2_, and then without ATP and MgCl_2_; buffer 3—200 mM KCl, 30 mM HEPES at pH 8, 5 mM K_2_HPO_4_/KH_2_PO_4_ at pH 8, 8% glycerol, 1 mM DTT and 0.5 mM EDTA. REV1 was eluted from the column with buffer 3 supplemented with 30 mM reduced glutathione at pH 8.0.

All of the five to six fractions containing REV1 were pooled and rhinoviral 3C protease was used to cleave the GST tag overnight at 4 °C. The next day, the protein was diluted with KCl-free buffer to a final concentration of 120 mM KCl and purified on the heparin–sepharose column (1 mL HiTrap Heparin HP, GE Healthcare) using the Akta Start (Cytiva, Marlborough, MA, USA). REV1 was eluted by a step gradient of elution buffer (600 mM KCl, 30 mM HEPES at pH 7.4, 5 mM K_2_HPO_4_/KH_2_PO_4_ at pH 7.4, 8% glycerol, 1 mM DTT, 0.005% NP-40 and 0.025% Tween20), and then KCl was increased to 800 and then to 1000 mM. Fractions containing REV1, observed by protein electrophoresis, were pooled and subjected to dialysis against 200 volumes of buffer 1 (400 mM KCl, 30 mM HEPES at pH 7.4, 5 mM K_2_HPO_4_/KH_2_PO_4_ at pH 7.4, 20% glycerol, 1 mM DTT, 0.005% NP40, and 0.025% Tween20) and then buffer 2 (storage buffer) (200 mM KCl, 30 mM HEPES at pH 7.4, 5 mM K_2_HPO_4_/KH_2_PO_4_ at pH 7.4, 20% glycerol, 1 mM DTT, 0.005% NP40 and 0.025% Tween20). The protein preparation was separated into 20–40 µL aliquots, frozen in liquid nitrogen and stored at −80 °C.
